# Identification and Characterization of the *Trypanosoma cruzi* B-cell Superantigen Tc24

**DOI:** 10.4269/ajtmh.15-0438

**Published:** 2016-01-06

**Authors:** Sarah M. Gunter, Kathryn M. Jones, Bin Zhan, Heather T. Essigmann, Kristy O. Murray, Melissa N. Garcia, Rodion Gorchakov, Maria Elena Bottazzi, Peter J. Hotez, Eric L. Brown

**Affiliations:** Center for Infectious Diseases, University of Texas School of Public Health, Houston, Texas; Baylor College of Medicine, National School of Tropical Medicine, Section of Tropical Medicine, Houston, Texas

## Abstract

*Trypanosoma cruzi* causes life-long disease after infection and leads to cardiac disease in 30% of infected individuals. After infection, the parasites are readily detectable in the blood during the first few days before disseminating to infect numerous cell types. Preliminary data suggested that the Tc24 protein that localizes to the *T. cruzi* membrane during all life stages possesses B-cell superantigenic properties. These antigens facilitate immune escape by interfering with antibody-mediated responses, particularly the avoidance of catalytic antibodies. These antibodies are an innate host defense mechanism present in the naive repertoire, and catalytic antibody–antigen binding results in hydrolysis of the target. We tested the B-cell superantigenic properties of Tc24 by comparing the degree of Tc24 hydrolysis by IgM purified from either Tc24 unexposed or exposed mice and humans. Respective samples were subjected to sodium dodecyl sulfate polyacrylamide gel electrophoresis, silver stained, and the degree of hydrolysis was measured. Data presented in this report suggest that the *T. cruzi* Tc24 is a B-cell superantigen based on the observations that 1) Tc24 was hydrolyzed by IgM present in serum of unexposed mice and humans and 2) exposure to Tc24 eliminated catalytic activity as early as 4 days after *T. cruzi* infection.

## Introduction

Chagas disease is a parasitic infection caused by the hemoflagellate protozoan *Trypanosoma cruzi*. There are an estimated 8–9 million prevalent human cases worldwide^1^ with an estimated 300,000–1 million infections in the United States.^2^ This vector-borne parasite is transmitted to humans and a wide variety of other mammalian hosts by the triatomine bug found throughout South and Central America, Mexico, and portions of the southern United States.^3^ Infection occurs when the parasite is released in the feces of a feeding triatomine and introduced into the host at mucosal surfaces or via skin abrasions. Less commonly, *T. cruzi* is transmitted congenitally, through blood transfusion, transplantation, or from consumption of contaminated food or beverages.^3^ Once inside a mammalian host, *T. cruzi* disseminates systemically and parasitic nests are most commonly found in the tissues of the heart and digestive tract.

Acute Chagas disease can be asymptomatic, but is often characterized by the appearance of a chagoma, an inflammatory nodule at the site of inoculation, followed by early clinical manifestations that can range from asymptomatic to general fever and facial edema in healthy persons. In immunosuppressed persons, symptoms can range from myocarditis, hepatomegaly, splenomegaly, and meningoencephalitis that occasionally can result in death.^4^ After the acute phase, the majority of people enter an indeterminate stage that is largely asymptomatic, but approximately 30% will develop cardiomyopathy or more rarely megacolon or megaesophagus. The current etiological treatments for Chagas disease, nifurtimox or benznidazole, are not Food and Drug Administration-approved medications and are associated with several severe adverse effects.^5^,^6^ Moreover, neither nifurtimox nor benznidazole reverse existing pathology.^5^,^6^ Because of the limited efficacy of available medications, a vaccine or novel therapeutic approach would be cost effective and benefit the prevention and treatment of Chagas disease.^7^,^8^ A better understanding of host/pathogen interactions including immune evasion strategies used by *T. cruzi* would facilitate these efforts.

Antibodies that develop as a consequence of antigen exposure and affinity maturation bind microbial antigens noncovalently and are central to immune defense against microbial pathogens. Of the immunoglobulins, IgM and homologous molecules were the first selected evolutionarily and the first to develop ontogenetically after exposure to antigen.^9^ IgM production by germ line–encoded immunoglobulin genes combines conserved evolutionary memory with effective effector functions in the absence of somatic hypermutation and develop in germ-free and antigen-free mice and represent an innate, first line of defense.^9^ Some of these IgM molecules are also catalytic antibodies that are capable of hydrolyzing target antigens because of the presence of specific amino acid sequences (e.g., the catalytic triad comprises Ser27a, His93, and Asp1) encoded by variable region germ line antibody genes.^10^,^11^ Because of their innate production and lack of somatic hypermutation, IgMs possess the most efficient antigen-specific catalytic activity (IgM > IgA > IgG).^12^

The naive antibody repertoire present in humans is derived from a large pool of B cells expressing diverse B-cell receptors (BCRs) generated by approximately 500 different germ line genes encoding the V_L_/V_H_, diversity, and joining segments that also hold the potential of generating thousands of antibodies of various classes (e.g., IgM, IgG, and IgA); each with a unique antigen-binding specificities.^13^ The combinational IgM repertoire derived from germ line V, D, and J segments can encode approximately 4 × 10^−9^ V_L_–V_H_ domain pairs and does not include expansion due to junctional diversification.^13^ This naive or natural antibody pool in both humans and animals has been shown to possess catalytic activities that range from promiscuous, that is, sequence-independent recognition of peptides to the hydrolysis of specific target antigens resulting from specific, noncovalent antigen recognition mediated by a serine protease mechanism.^10^

Nucleophilic sites encoded by germ line V genes (without the need for antigen stimulation through the BCR) and selected over millions of years are universally expressed by antibodies resulting in promiscuous catalytic antibody activity,^14^ suggesting that this innate (promiscuous) activity provides a homeostatic function.^15^ For example, high and low levels of promiscuous catalytic activity has been linked to decreased and increased risk of death in patients with sepsis, respectively.^16^,^17^ In contrast to antibodies with promiscuous activity, antibodies with noncovalent antigen-binding specificities to respective antigens are endowed with the ability of hydrolyzing specific targets. That is, although different antibodies may have the potential for enzymatic activity, lysis of specific targets is the result of initial antigen specific, noncovalent binding followed by target hydrolysis.^10^ Because of the ability of catalytic antibodies to hydrolyze and inactivate their targets, their potential role in neutralizing microbial antigens is significant; however, their role in protection against microbial pathogens remains largely unexplored.

A subset of antigens have been defined as B-cell superantigens (BC-SAgs) because 1) antibodies present in the naive repertoire encoded by germ line sequences have specificity for these antigens, for example, staphylococcal protein A and the extracellular fibrinogen-binding protein, and human immunodeficiency virus (HIV) gp120^10^,^18^–^21^ and 2) exposure to BC-SAgs results in the depletion of the B-cell subsets secreting antibodies with BC-SAg hydrolyzing potential, leaving a “hole” in the catalytic repertoire.^10^,^11^ We hypothesize that BC-SAgs evolved in part to counter catalytic antibody-mediated hydrolysis of target antigens.

As described above, the HIV gp120 glycoprotein represents one example of a BC-SAg that can be hydrolyzed by IgM purified from uninfected people^22^ and it's hydrolysis is lost after BC-SAg exposure as a consequence of clonal B-cell depletion.^22^ As described for HIV/acquired immune deficiency syndrome, *T. cruzi* may also produce a potential BC-SAg. For example, Tc24 is a member of the excretory/secretory antigen family^23^ that localizes to the membrane during all life stages and is primarily expressed at the flagellar pocket.^24^,^25^ B cells exposed to Tc24 have been shown to clonally expand both in vivo and in vitro^26^ and the immunoglobulin response elicited by Tc24 is nonspecific and composed primarily of IgM, similar to what occurs after B-cell stimulation by mitogens. In addition, injection of Tc24 into athymic mice suggested that the observed B-cell activation occurred independent of T cell help.^24^ Data presented in this report suggest that Tc24 exhibits BC-SAg properties based on the observation that exposure to Tc24 via either antigen injection or infection with *T. cruzi* significantly reduced IgM-mediated cleavage of Tc24.

## Materials and Methods

### Recombinant proteins.

Recombinant Tc24 was expressed in *Escherichia coli* BL21(DE3) by cloning an *E. coli* codon-optimized DNA coding for full-length Yucatan strain *T. cruzi* 24 kDa flagellar calcium-binding protein (Tc-24E-Yucatan, amino acids 1–211) in frame into pET41a vector between *Nde*I and *Eco*RI sites. Na-ASP-2 (*Ancylostoma*-secreted protein) was cloned from human hookworm *Necator americanus* and expressed as a recombinant protein in *Pichia pastoris* X-33 as described previously.^27^ The recombinant *Staphylococcus aureus* S component of the Panton–Valentine bicomponent pore-forming leukotoxin (LukS-PV) and the enteroaggregative *E. coli* strain O42 dispersin protein have been described in previous work.^28^–^31^ Recombinant *S. aureus* toxic shock syndrome toxin (TSST) was purchased from IBT Bioservices (Gaithersburg, MD).

### Parasites and mice.

*Trypanosoma cruzi* CL strain expressing tdTomato (CL-tdTomato)^32^ was provided by Rick Tarleton (University of Georgia, GA) and trypomastigotes were maintained by continuous in vitro passage in C2C12 cells. *Trypanosoma cruzi* H1 strain previously isolated from a human case in Yucatan, Mexico^33^ was provided by Eric Dumonteil (Universidad Autónoma de Yucatán) and maintained by serial passage in Balb/c mice. Female Balb/c mice, 6–8 weeks old, were obtained from Taconic Biosciences (Hudson, NY).

### Immunizations and infections.

Naive mice were injected twice, 2 weeks apart with 25 μg recombinant Tc24 protein adsorbed to 200 μg Alhydrogel^®^ (Brenntag Biosector, Frederikssund, Denmark) (referred to as alum for the remainder of the report), or alum only. Blood was collected 2 weeks after the second injection by cardiac puncture under anesthesia (150 mg/kg ketamine/15 mg xylazine). Pooled sera from noninfected and noninjected Balb/c, C57BL/6, CD-1, and non-Swiss albino mice were also obtained from Innovative Research (Novi, MI).

For acute infection studies, naive mice were infected with 500 blood-form trypomastigotes of *T. cruzi* H1 and killed at 4, 7, 21, 28, or 49 days after infection. For chronic infection studies, mice were infected with 1,000 tissue culture trypomastigotes of *T. cruzi* CL-tdTomato by intraperitoneal injection and killed at 166 and 238 days after infection. At the designated time points, mice were exsanguinated by cardiac puncture under anesthesia as described above. All mouse studies were performed in compliance with the National Institutes of Health Guide for the Care and Use of Laboratory Animals and were approved by the Baylor College of Medicine Institutional Animal Care and Use Committee.

### Human samples.

Written informed consent was obtained before human serum samples were collected and the study was approved by the Baylor College of Medicine Institutional Review Board. Blood samples were collected from individuals identified as potentially Chagas positive by the Gulf Coast Regional Blood Bank (Houston, TX), as previously described.^34^ Human samples were divided based on serology into either Chagas-positive or Chagas-negative controls. Chagas positive were individuals that tested positive for Chagas on six different diagnostic tests: ortho *T. cruzi* enzyme-linked immunosorbent assay (ELISA) (Ortho-Clinical Diagnostics, Rochester, NY), radioimmunoprecipitation assay, Stat-Pak (Chembio Diagnostic Systems, Medford, NY), DPP (ChemBio Diagnostic Systems), Chagatest (Wiener Laboratories, Santa Fe, Argentina), and immunofluorescence antibody assays. Chagas-negative individuals were defined as testing negative for Chagas using all serological tests described above. Serum was isolated from blood samples of 13 Chagas-positive individuals and five Chagas-negative controls. Chagas-positive individuals were further divided by the presumed region where the infection was acquired. Infections acquired in the United States were defined by no significant history of travel to endemic regions or having received blood or tissue translplants.^34^ To rule out congenital transmission, these individuals and their mothers also had to be born in the United States. Participants not matching the definitions for locally acquired infections were considered to have been infected in an endemic area as described previously.^35^

### IgM and IgG purification.

IgM and IgG antibodies were purified from mouse or human serum samples using CaptureSelect^™^ Affinity Matrix from Life Technologies (Grand Island, NY). To isolate IgM, serum (≤ 1 mL) was applied to columns containing 400 μL of IgM CaptureSelect IgM Affinity Matrix resin and the flow through was collected and stored at −80°C until used for IgG purification or applied directly to CaptureSelect IgG Affinity Matrix resin. IgM and IgG isotypes were collected as specified by the manufacturer. Briefly, columns were washed with 5 column volumes (CVs) of phosphate-buffered saline (PBS). IgM or IgG were eluted in 5 CV of 0.1 M glycine (pH 3.0) into a tube containing 0.1 CV of 1 M Tris, pH 8.0. The elution fraction was then dialyzed against PBS. Immunoglobulin concentrations were determined using a micro BCA kit (Pierce, Rockford, IL). Electrophoretic homogeneity of antibody preparations was assessed by subjecting IgM and IgG purified from the serum of mice or humans in respective groups to either reducing or nonreducing 15% sodium dodecyl sulfate polyacrylamide gel electrophoresis (SDS-PAGE) followed by either silver staining or immunoblotting. Human IgM preparations were blotted onto nitrocellulose paper and probed with goat antihuman μ-chain–specific peroxidase conjugated (Sigma-Aldrich Corporation, St. Louis, MO), goat antihuman κ light chain–specific peroxidase conjugated (Sigma-Aldrich Corporation), or goat antihuman *λ* light chain–specific peroxidase conjugated (Sigma-Aldrich Corporation) antibodies. Mouse IgM or IgG preparations were blotted onto nitrocellulose paper and probed with rabbit anti-mouse κ light chain (MP Biomedicals, Santa Ana, CA) and band visualization was carried out using a goat anti-rabbit alkaline phosphatase (AP) antibody (Sigma-Aldrich Corporation).

### Proteolysis assays.

IgM- or IgG-mediated hydrolysis of Tc24 (and control proteins) was determined by incubating 15 μL of respective purified immunoglobulin preparations with 5 μL of 10.4 μM Tc24 in PBS with 4 mM 3-[(3-Cholamidopropyl)dimethylammonio]-1-propanesulfonate and 268 μg/mL gelatin at 37°C with shaking.^14^,^19^ Reactions were stopped at various time points by adding 4× nonreducing SDS-PAGE loading buffer (Bio-Rad, Hercules, CA). Hydrolysis of target antigens was conducted using antibody concentrations ranging between 14 and 135 μg/mL (diluted in PBS). Hydrolysis rates were determined and expressed as percentage Tc24 hydrolysis using the following equation: (1−[s−Ab]/[s−BC]) × 100. In this equation [s−Ab] and [s−BC] represent the intensity of the intact protein band for the antibody-containing reaction and the control reaction containing only buffer, respectively. Band intensities were determined using ImageJ software (Bethesda, MD).

To determine kinetics of the hydrolysis reaction, hydrolysis was measured at increasing concentrations of Tc24 and data fit to the Michaelis–Menten equation. The ability of protease inhibitors to inhibit respective hydrolysis reactions was assessed by adding either a serine protease inhibitor, 4-(2-Aminoethyl)benzenesulfonyl fluoride hydrochloride (AEBSF) (1 mM); a cysteine protease inhibitor, leupeptin (50 μM); or a metalloprotease inhibitor, ethylenediaminetetraacetic acid (EDTA) (2 mM).

### Binding assays.

Binding of IgG antibody to immobilized Tc24 was measured in triplicate using an ELISA as previously descried.^36^ In brief, bound mouse IgG was detected using a goat anti-mouse γ-chain specific AP-conjugated antibody. The reaction was developed by adding 100 μL of 1 mg/mL Sigma 104 Phosphatase substrate (Sigma-Aldrich Corporation) dissolved in 1 M diethanolamine, 0.5 mM MgCl_2_, pH 9.8 for 1 hour. The optical density was measured at 405 nm using a microplate reader (Molecular Devices, Menlo Park, CA).

## Results

### IgM-mediated hydrolysis of Tc24.

**Figure 1.**
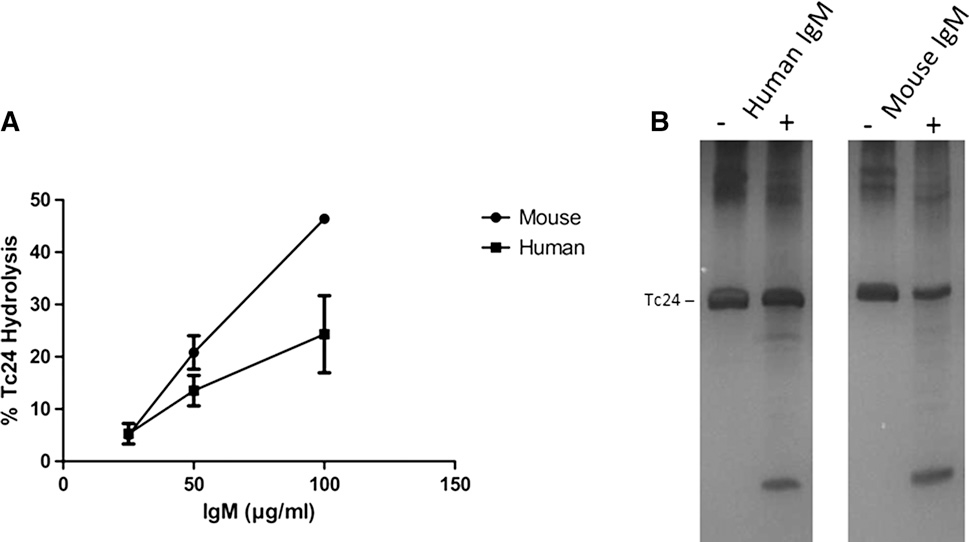
Hydrolysis of Tc24 by IgM purified from unexposed mice and humans. IgM was pooled from the sera of naive Balb/c mice and from the sera of five individuals that tested negative for Chagas and Tc24 hydrolysis was assessed over a period of 48 hours. (**A**) Dose-dependent hydrolysis of Tc24 by mouse or human IgM (0, 25, 50, and 100 μg/mL). (**B**) Representative silver-stained gel demonstrating hydrolysis of Tc24 (2.6 μM) by either human or mouse IgM (100 μg/mL). Parent band depletion was measured using ImageJ.

A hallmark of BC-SAgs is their susceptibility to hydrolysis by catalytic antibodies present in the naive repertoire. Because IgM antibodies are derived from germ line immunoglobulin sequences with the potential of mediating hydrolysis of respective BC-SAg targets and previously demonstrated to possess the highest levels of enzymatic activity,^14^ IgM was purified from pooled mouse sera and from the sera of five healthy human negative–control donors. Pooled, polyclonal IgM preparations were subsequently incubated in the presence of Tc24 and hydrolysis determined by subjecting samples to SDS-PAGE and measuring Tc24 depletion (Figure 1A

and B).

### IgM purity, specificity, and nature of the enzymatic reaction.

**Figure 2.**
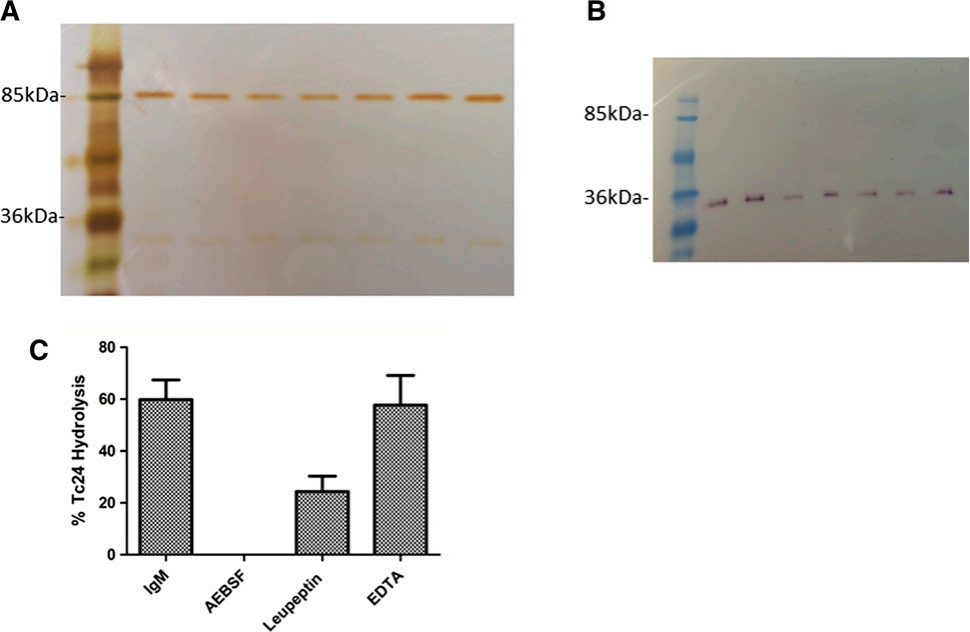
IgM purity, rate, and mechanism of hydrolysis. IgM from seven mice randomly selected from respective treatment groups was subjected to sodium dodecyl sulfate polyacrylamide gel electrophoresis (SDS-PAGE) and either (**A**) silver-stained or (**B**) transferred to nitrocellulose paper and probed with an alkaline phosphatase-conjugated rabbit anti-mouse kappa chain specific monoclonal antibody. (**C**) The effect of various inhibitors on IgM-mediated hydrolysis was tested by incubating 45 μg/mL IgM purified from alum-only injected mouse serum with 2.6 μM Tc24 for 48 hours at 37°C with shaking in the presence or absence of a serine protease inhibitor, AEBSF (1 mM); a cysteine protease inhibitor, leupeptin (50 μM); or a metalloprotease inhibitor, Ethylenediaminetetraacetic acid (2 mM); and then subjected to SDS-PAGE using 15% Tris-Gly gels. Percent cleavage calculated using ImageJ.

The purity of each IgM preparation used was confirmed by both silver staining and immunoblot analyses demonstrating that respective preparations were electrophoretically homogenous (Figure 2A

and B). Purity was assessed for all IgM preparations used in subsequent hydrolysis experiments (data not shown).

We observed saturation kinetics consistent with the Michaelis–Menten equation (Table 1). The apparent 5.11 × 10^3^ (M^−1^ min^−1^) *k*_cat_/K_M_ is similar in enzyme efficiency to previously reported catalytic antibodies with reactivity against different substrates.^19^,^37^,^38^ Presence of the serine protease inhibitors, AEBSF and leupeptin (also a cysteine and threonine protease inhibitor), reduced IgM-mediated hydrolysis of Tc24 compared with no inhibition observed for the metalloprotease inhibitor EDTA. The diminished inhibitory effect of leupeptin relative to that of AEBSF was not surprising as leupeptin has been shown be a less effective serine protease inhibitor, and in one study failed completely to inhibit serum IgA-mediated hydrolysis.^39^,^40^ These experiments demonstrated that catalytic antibody–mediated hydrolysis of Tc24 occurred via a serine protease mechanism as previously described for other catalytic antibody-mediated hydrolysis reactions (Figure 2C).^19^

**Figure 3.**
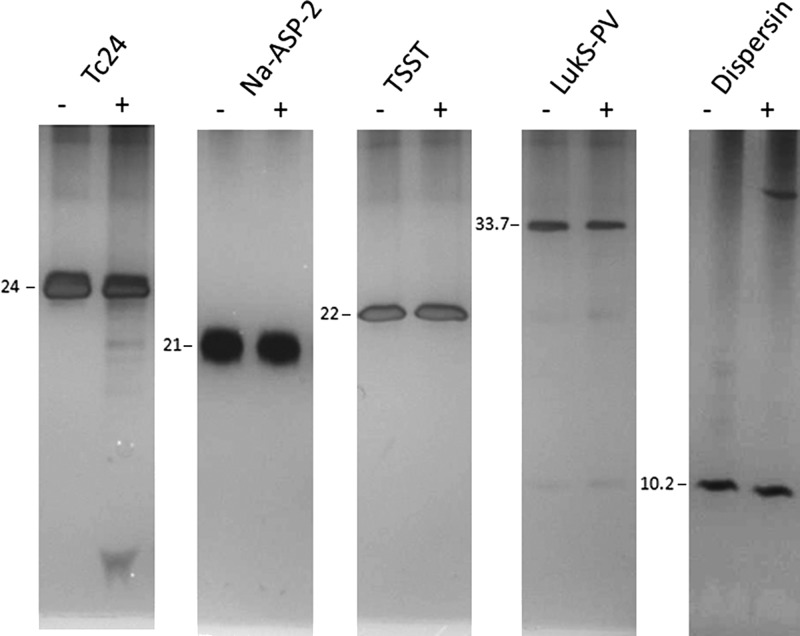
Specificity of IgM-mediated hydrolysis. IgM (45 μg/mL) purified from alum-only injected mouse serum was incubated with 2.6 μM of either Tc24, Na-ASP-2, Panton–Valentine bicomponent pore-forming leukotoxin (LukS-PV), toxic shock syndrome toxin (TSST), or dispersin for 24 hours at 37°C with shaking. Samples were then subjected to sodium dodecyl sulfate polyacrylamide gel electrophoresis using 15% Tris-Gly gels and silver-stained.

The specificity of the IgM-mediated hydrolysis reaction for Tc24 was demonstrated by incubating non-BC-SAg recombinant proteins generated from various pathogens including *N. americanus* (Na-ASP-2), a major protein released by infective hookworm larvae, *S. aureus* (LukS-PV and TSST), and *E. coli* (dispersin). Only Tc24 was hydrolyzed by IgM purified from alum-only immunized animals (Figure 3

).

### Tc24 injection reduces IgM-mediated hydrolysis of Tc24.

**Figure 4.**
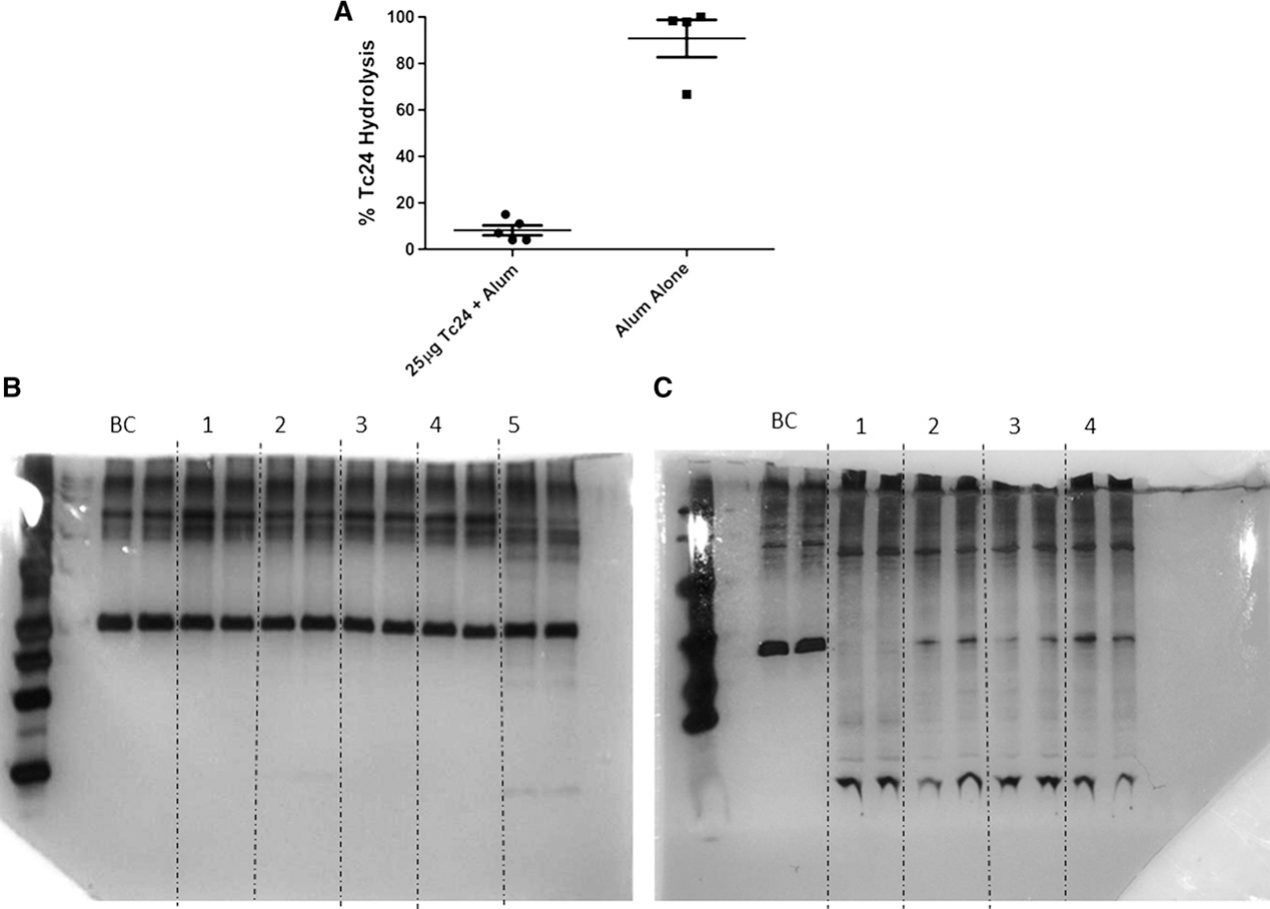
Impact of injection with Tc24 on IgM-mediated hydrolysis. Balb/c mice were injected with either 25 μg Tc24 + 200 μg alum or 200 μg alum only (control). (**A**) IgM (50 μg/mL) from the Tc24 injected group and from the alum injected group was incubated with 2.6 μM Tc24 for 40 hours at 37°C with shaking, and then subjected to sodium dodecyl sulfate polyacrylamide gel electrophoresis using 15% Tris-Gly gels. Percent cleavage was calculated using ImageJ. Representative silver-stained 15% Tris-Gly gels of Tc24 hydrolysis by IgM purified from either (**B**) 25 μg Tc24 + 200 μg alum injected mice or (**C**) alum-only injected mice. Numbers indicate individual IgM samples purified from mice in respective groups tested in Tc24 hydrolysis assays, in duplicate. BC = buffer control (contains all reagents except for purified IgM). *P < 0.0001 using the unpaired, two-tailed *t* test.

Hydrolysis of respective antigens by antibodies present in the naive repertoire is not sufficient to define a BC-SAg. A second criterion requisite of BC-SAgs is their ability to reduce catalytic activity specific to that BC-SAg after exposure. This is accomplished as a consequence of the signals resulting from the antibody–BC-SAg interaction, that is, B-cell clones capable of binding and hydrolyzing respective BC-SAgs are deleted from the repertoire.^19^,^22^ This criterion was assessed by measuring hydrolysis of Tc24 after incubation with IgM preparations purified from mice injected with Tc24 in the presence of alum, or from mice injected with 200 μg alum only (negative control) (Figure 4A

). The IgM concentration tested was 50 μg/mL for IgMs purified from Tc24 injected or alum-only injected mice. This concentrations is 40 times less than physiologic IgM concentrations. Representative silver-stained gels demonstrate hydrolysis of the parent Tc24 band by IgM purified from alum-only injected mice (Figure 4C) compared with hydrolysis carried out by IgM purified from mice injected with 25 μg Tc24 + 200 μg alum (Figure 4B). These data suggest that the IgM pool purified from mice exposed to Tc24 was devoid of a subset of IgM molecules present in the other IgM preparations that mediated Tc24 hydrolysis.

### *Trypanosoma cruzi* infection reduces IgM-mediated hydrolysis of Tc24.

**Figure 5.**
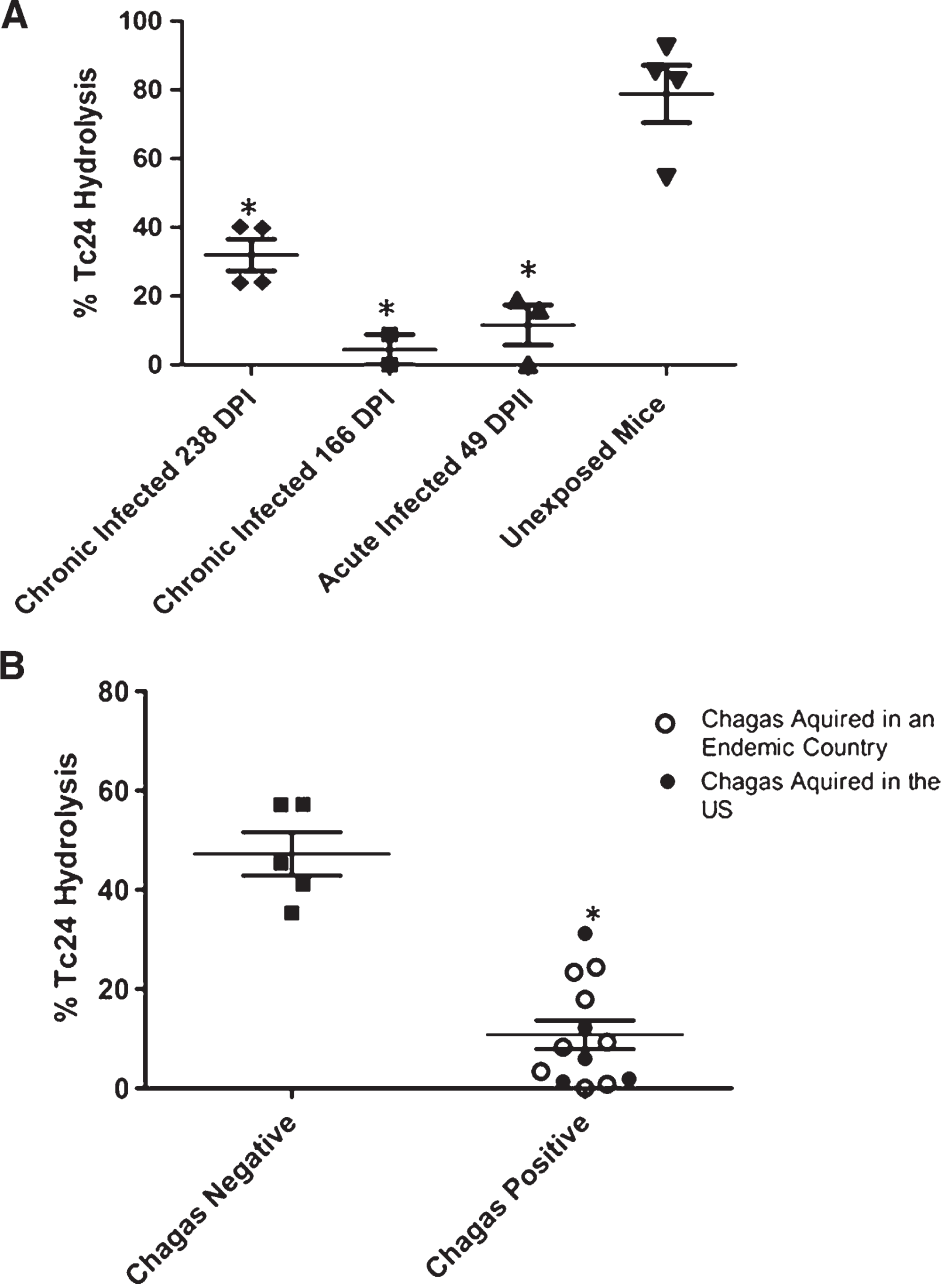
Effect of *Trypanosoma cruzi* infection on IgM-mediated hydrolysis of Tc24. (**A**) Balb/c mice were infected with either H1 or CL-tdTomato strains of *T. cruzi* and allowed to progress to either acute or chronic disease states. IgM (30 μg/mL) purified from infected or uninfected mice was incubated with 2.6 μM Tc24 at 37°C with shaking and then subjected to sodium dodecyl sulfate polyacrylamide gel electrophoresis (SDS-PAGE) using 15% Tris-Gly gels. Percent cleavage was calculated using ImageJ. (**B**) IgM (45 μg/mL) purified from human serum samples collected from individuals with either Chagas positive or Chagas negative serology tests was incubated with 2.6 μM Tc24 for 24 hours at 37°C with shaking and then subjected to SDS-PAGE using 15% Tris-Gly gels. Percent cleavage calculated using ImageJ. DPI = days postinfection. *P < 0.005 using the unpaired, two-tailed *t* test.

To examine the impact of *T. cruzi* infection on the hydrolysis of Tc24, IgM was purified from the serum of either *T. cruzi-*infected mice or from humans diagnosed with Chagas disease on the basis of serology. As observed after immunization with Tc24, *T. cruzi* infection also resulted in a significant reduction in Tc24 hydrolysis by IgM preparations purified from the respective groups (Figure 5A

and B).

**Figure 6.**
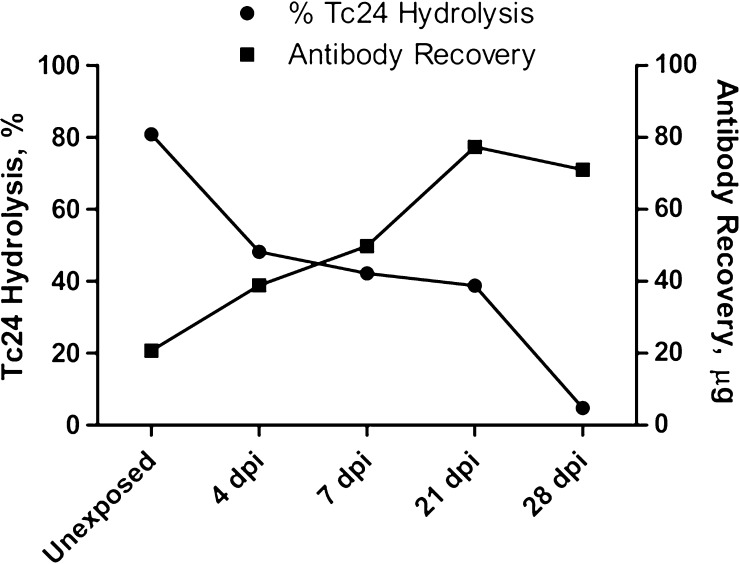
Temporal effects of antigen exposure on catalytic activity. Mice were infected with *Trypanosoma cruzi* by injection of 500 blood form trypomastigotes of *T. cruzi* H1 and individual mice were killed at 4, 7, 21, or 28 days postinfection. IgM (65 μg/mL) purified from individual mice killed at each time point was incubated with 2.6 μM Tc24 for 72 hours at 37°C with shaking and subjected to sodium dodecyl sulfate polyacrylamide gel electrophoresis using 15% Tris-Gly gels. Percent cleavage calculated using ImageJ. IgM recovery from individual mice was determined using Micro BCA kit.

We next assessed the temporal effects of Tc24 exposure on catalytic activity after infection with *T. cruzi* H1 strain. IgM was purified from individual mice that were killed at 4, 7, 21, or 28 days postinfection and tested in Tc24 hydrolysis assays. These data demonstrated a significant and time-dependent reduction in hydrolytic activity detectable as early as day 4 postinfection (Figure 6

). This temporal reduction in catalytic activity was associated with a parallel increase in the IgM concentration after infection suggesting that the reduction in catalytic activity is independent of the ability to generate an IgM response (Figure 6).

## Discussion

Data presented in this report demonstrated that Tc24 is a BC-SAg based on the observations that: 1) Tc24 was hydrolyzed by IgM purified from mice and humans not previously exposed to Tc24 by either injection or *T. cruzi* infection and 2) after exposure to Tc24, the subset of IgM antibodies mediating Tc24 hydrolysis is absent similar to what has been described for other BC-SAgs.^19^,^37^ Responses to BC-SAgs (i.e., reduction in catalytic activity) are opposite to responses generated after exposure to conventional antigens, which are recognized by complementarity determining regions leading to class-switching and affinity maturation.^41^ Antibodies with catalytic potential are an innate immune defense mechanism not associated with adaptive immunity that likely evolved as a means of hydrolyzing antigenic targets. BC-SAgs represent a microbial response to this host defense mechanism that is important to pathogens such as *T. cruzi* that spend a period of time in the blood stream.

We observed significant hydrolysis of Tc24 at concentrations of IgM approximately 40 times less than the 2 mg/mL present in serum. These data suggested that the presence of catalytic IgM in the preimmune serum may provide some degree of protection against acute forms of disease if the catalytic activity were to remain present long enough to prevent *T. cruzi* replication and dissemination. On the basis of the observation that catalytic activity was significantly reduced by day-4 postinfection, it is likely that protective catalytic responses would be marginal at best, which may explain why catalytic responses are not protective in the context of acute *T. cruzi* infections. That IgM plays an important role during the early stages of infection and provides some level of innate protection is supported by the observation that rats deficient in IgM had an increased susceptibility to acute infection.^42^ What level of protection would be achieved if catalytic activity were not abolished remains unknown at this time. Therefore, developing vaccine formulations that stimulate rather than reduce catalytic activity against Tc24 may potentially result in a robust immune response with the potential of controlling the initial infection.

Another variable potentially affecting susceptibility to infection is heterogeneity of hydrolysis rates between individuals and species. This was tested by purifying IgM from different mouse strains and demonstrated that these strains possessed different hydrolysis rates (Supplemental Figure 2). At this juncture, it would be premature to speculate as to the significance of these differences in hydrolysis because there are conflicting results in the literature regarding what mouse strains are more or less resistant to *T. cruzi* infection.^43^–^46^ In addition, IgM-mediated Tc24 hydrolysis rates between Balb/c mice and humans were different. However, these differences may be a reflection of how the heterogeneity of the immune response affects acute and chronic disease progression.

The specificity of the Tc24 hydrolysis reaction was demonstrated by the ability of IgMs purified from non-Tc24-exposed mice to hydrolyze Tc24 but not other non-BC-SAgs, including Na-ASP-2 from *N. americanus*, TSST and LukS-PV from *S. aureus*, or dispersin from *E. coli* suggesting that the activity reported was Tc24 specific. Hydrolysis of Tc24 occurred via a serine-protease mechanism similar to previously described catalytic antibody-mediated hydrolysis reactions.^19^ Although exposure to BC-SAgs reduced catalytic activity, conventional IgG-mediated recognition of alternative epitopes on Tc24 was readily observed (Supplemental Figure 1A). However, no detectable hydrolysis of Tc24 by purified IgG from alum-injected mice was observed as previously demonstrated for other BC-SAgs (Supplemental Figure 1B).

We hypothesize that the B cells that produce Tc24 hydrolyzing IgM antibodies are either deleted or anerigized after exposure to Tc24 (either as a result of immunization or infection) and that suppression of catalytic responses is sustained throughout *T. cruzi* infections since Tc24 is secreted and expressed by all *T. cruzi* developmental stages. This impact to the IgM response appears to be limited to the B cells secreting catalytic IgM antibodies since the IgM concentration following *T. cruzi* infection increased, suggesting that IgMs with specificities to other targets may not play a significant role in controlling the infection and that any benefit conferred by the catalytic response would be during the early stages of infection (Figure 6). A Tc24-specific IgG binding (but not catalytic as described in the context of other BC-SAgs) response was also observed and was likely mounted in response to epitopes on Tc24 that are not superantigenic. “Silencing” of the anti-Tc24 catalytic response early in the infection process may represent a key immune escape mechanism that allows the parasite to survive in the bloodstream after infection and may leave the host more susceptible to subsequent infections since there is now a “hole” in the catalytic IgM repertoire. As the infection progresses and *T. cruzi* parasitizes intracellular niches, the presence of antibodies in general may not play as an important a role in containment of the organism compared with early in the infection process. Although, a robust IgG response develops (Supplemental Figure 1), we do not at this time know how this response impacted the early stages of infection.

This report is the first to describe a BC-SAg produced by a protozoan. In comparison with bacteria and viruses, protozoans and helminths are highly complex organisms and it is likely that Tc24 is the first of many BC-SAgs produced not only by *T. cruzi* but also other parasites with blood stages (e.g., Schistosomes and *Plasmodium* sp.). Data presented in this report suggest that the measurement and manipulation of catalytic antibody responses could be used to more effectively manage Chagas disease for which current diagnostic, treatment, and vaccine options remain limited.

## Supplementary Material

Supplemental Figures.

## Figures and Tables

**Table 1:** Antibody kinetics

Antibody	Apparent *K*_M_	*k*_cat_ (min^−1^) or apparent *V*_max_ (μM substrate/μM IgM/minute)
Mouse IgM	4.3 ± 3.9	0.022 ± 0.006

50 μg/mL IgM purified from alum-only vaccinated mouse serum was incubated with varying concentrations of Tc24 (2.6, 5.2, 10.4, and 20.8 μM) for 19 hours at 37°C with shaking and then subjected to sodium dodecyl sulfate polyacrylamide gel electrophoresis using 15% Tris-Gly gels. Percent cleavage calculated using ImageJ. Apparent *k*_cat_ and *K*_M_ values were calculated by fitting data to the Michaelis–Menten equation.
