# Supplementary material for: Identification and Characterization of the Trypanosoma cruzi B-cell Superantigen Tc24
Source: Am J Trop Med Hyg. 2016 Jan 6;94(1):114–21. doi: 10.4269/ajtmh.15-0438 (PMC4710414; doi:10.4269/ajtmh.15-0438)
Supplement: Supplementary file 1 [file SD2.pdf]

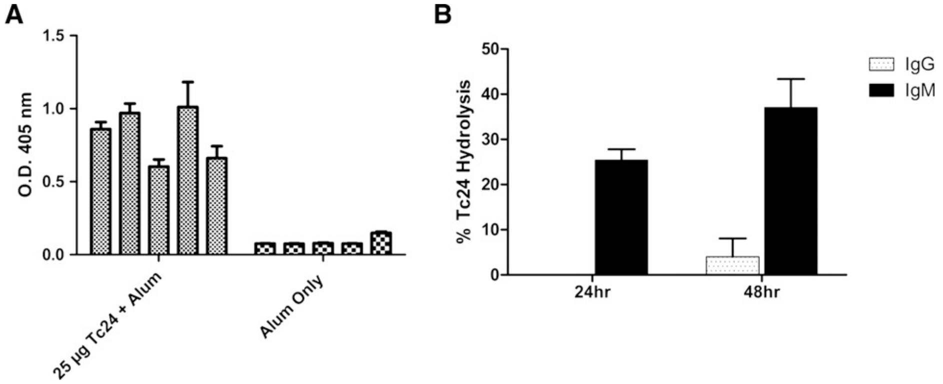

Supplemental Figure 1. Anti-Tc24 binding and hydrolysis by IgG. (**A**) Binding to immobilized Tc24 by IgG from Tc24-vaccinated mice and alum-only vaccinated mice was measured by enzyme-linked immunosorbent assay (each bar represents an individual mouse). A 1:500 dilution of the 1 mL flow through collected from the IgM purification step was used to assess IgG binding. (**B**) Hydrolysis of Tc24 by IgG was compared with IgM by incubating 14 μg/mL of either pooled IgG or IgM purified from alum-only vaccinated mice with 2.6 μM of Tc24 for either 24 or 48 hours at 37°C with shaking and then subjected to sodium dodecyl sulfate polyacrylamide gel electrophoresis using 15% Tris-Gly gels. Percent cleavage was calculated using ImageJ.

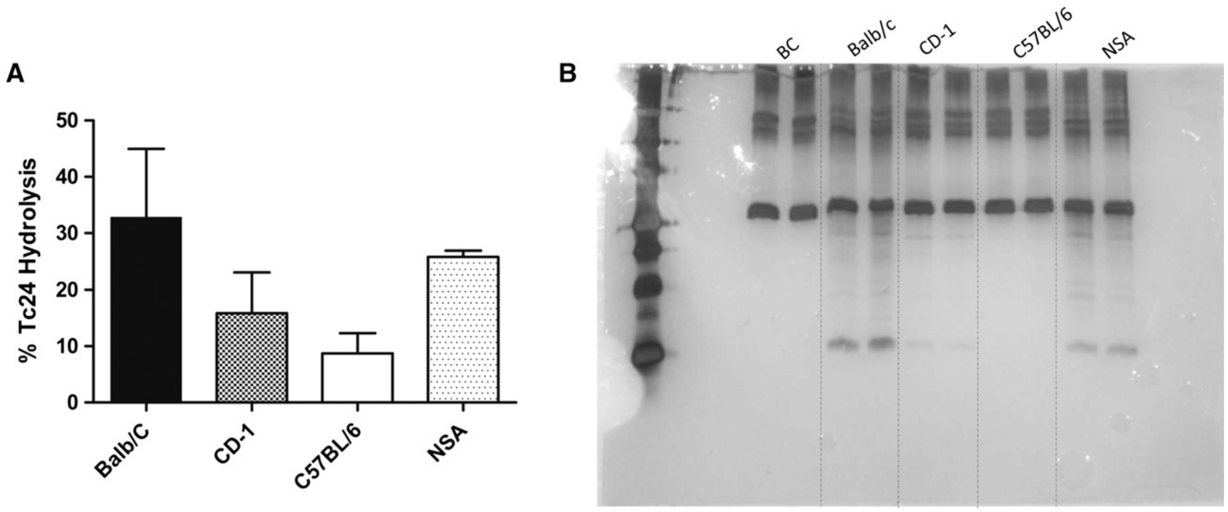

Supplemental Figure 2. IgM-mediated hydrolysis of Tc24 by different mouse strains. (**A**) 135 μg/mL IgM purified from either pooled Balb/c, CD-1, C57BL/6, or non-Swiss albino (NSA) mouse sera, respectively, was incubated with 2.6 μM Tc24 for 48 hours at 37°C with shaking and then subjected to sodium dodecyl sulfate polyacrylamide gel electrophoresis using 15% Tris-Gly gels. Percent cleavage was calculated using ImageJ. (**B**) Representative silver-stained 15% Tris-Gly gels of Tc24 hydrolysis by IgM purified from Balb/c, CD-1, C57BL/6, or NSA mice. Hydrolysis reactions were run in duplicate.
